# Supplementary figures and images for: EGFR-targeted affibody–polyIC polyplex kills EGFR-overexpressing cancer cells without activating the EGFR
Source: PLoS One. 2026 May 5;21(5):e0334584. doi: 10.1371/journal.pone.0334584 (PMC13143101; doi:10.1371/journal.pone.0334584)

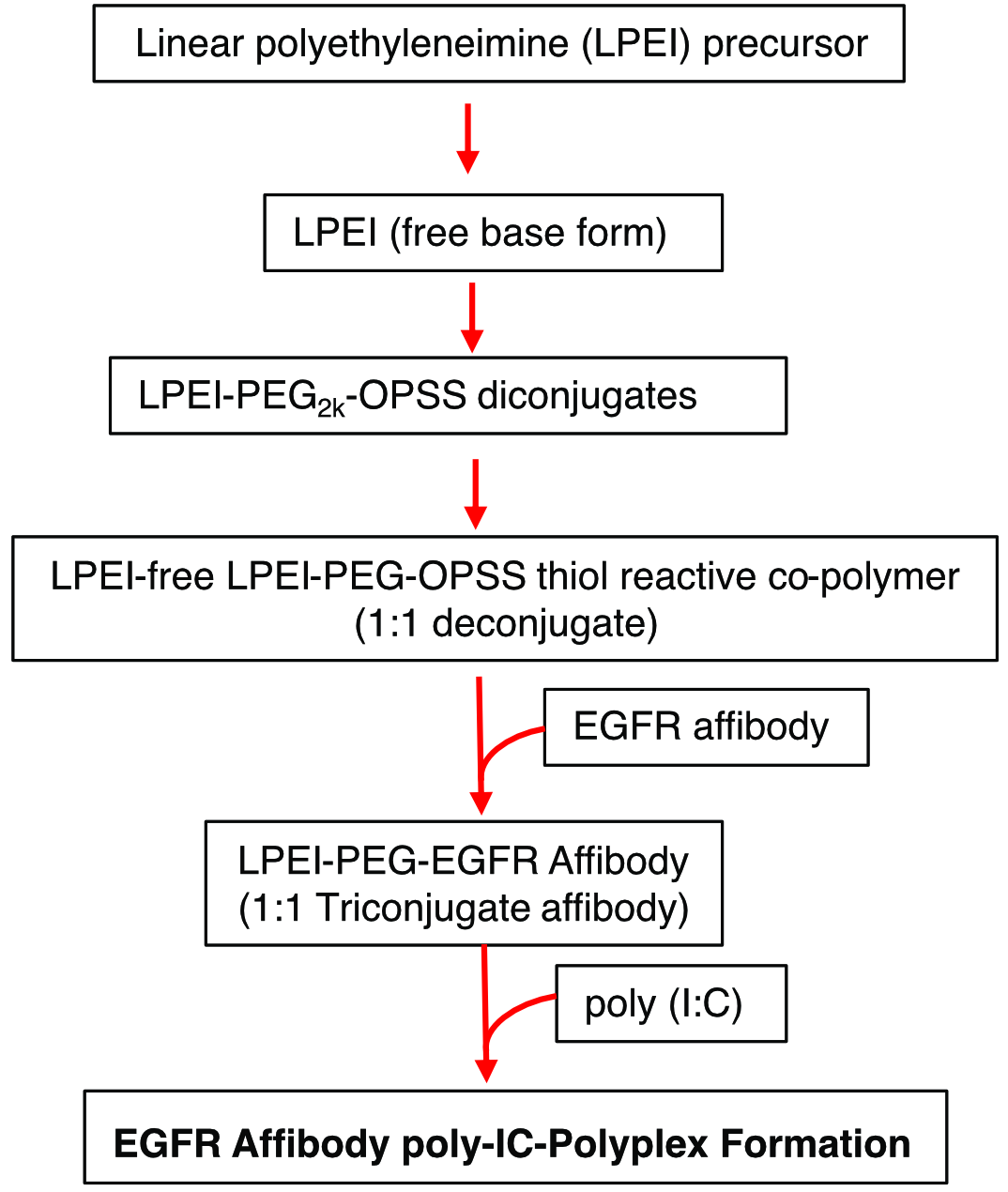

Supplement: S1 Fig — The details for each step of the synthesis are described in the Materials and methods section. (TIFF) [file pone.0334584.s001.tiff]

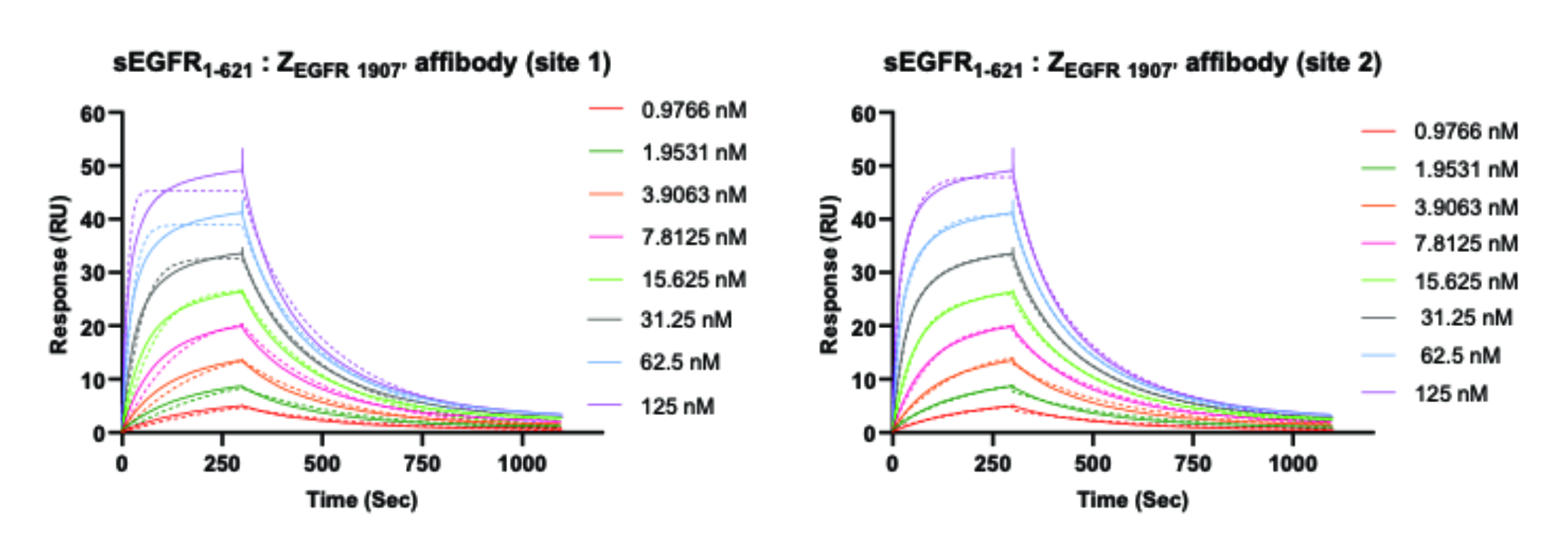

Supplement: S2 Fig — Analysis of binding data obtained for ZEGFR 1907’ affibody with hEGFR1–621 provides justifications for two sites fits model. Solid and dotted line graphs represent raw and fitted data respectively. (TIFF) [file pone.0334584.s002.tiff]

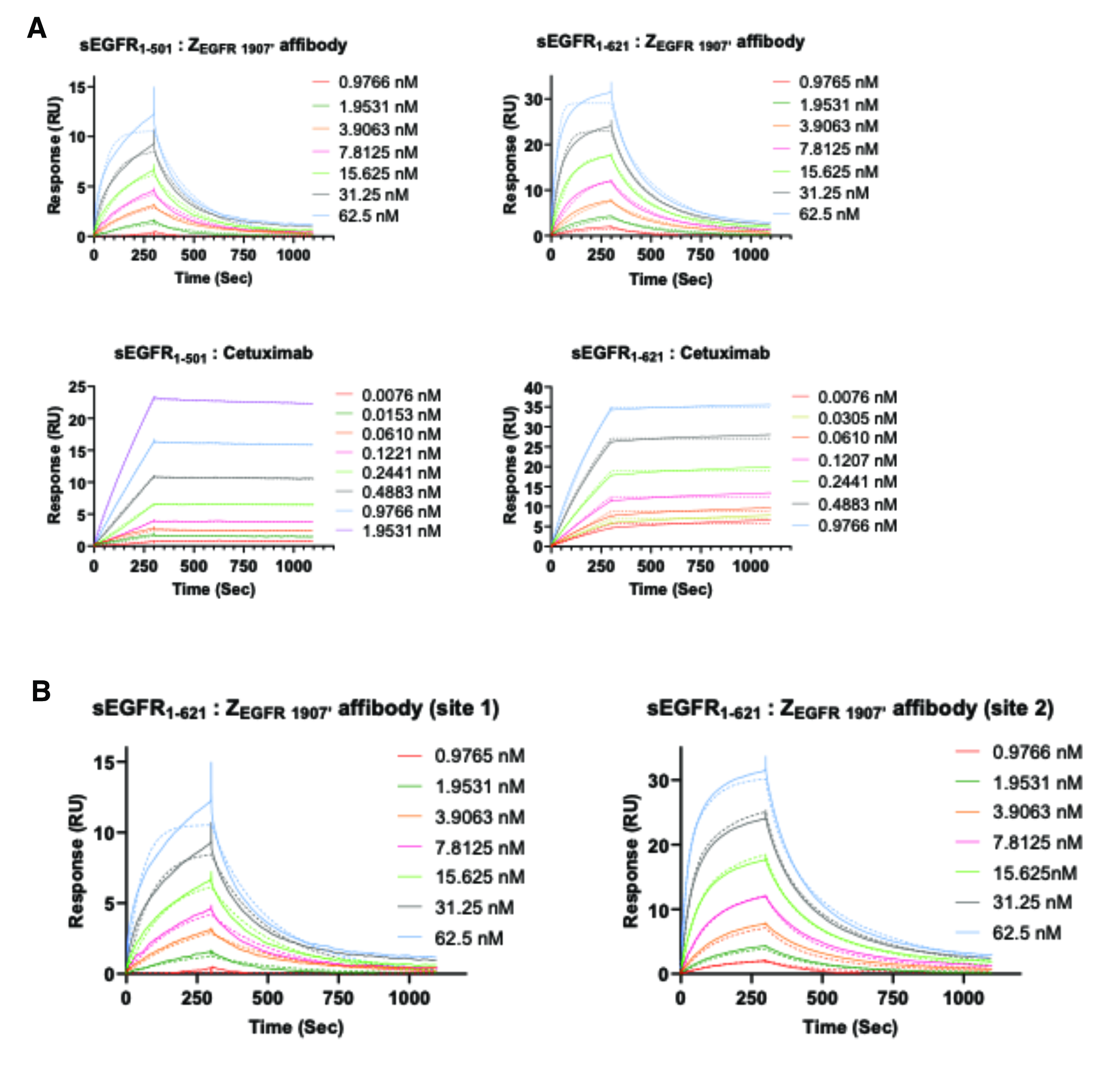

Supplement: S3 Fig — (A) Binding kinetics for the ZEGFR 1907’ affibody and Cetuximab interacting with hEGFR1–501 and hEGFR1–621, measured using the Biacore S200 and used to determine Kd values. (B) Analysis of binding data obtained for ZEGFR 1907’ affibody with hEGFR1–621 provides justifications for two sites fits model. Solid and dotted line graphs represent raw and fitted data respectively. (TIFF) [file pone.0334584.s003.tiff]

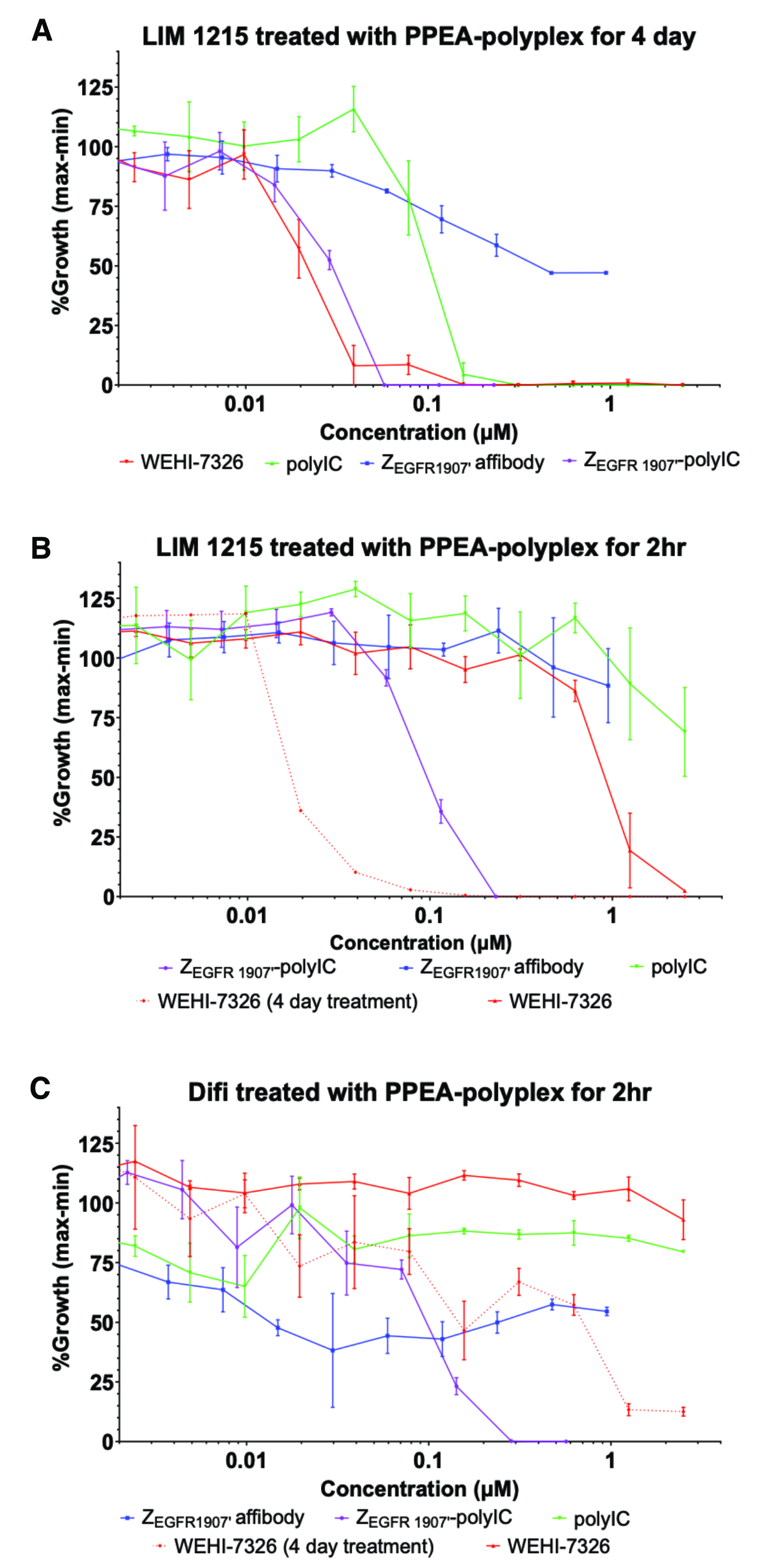

Supplement: S4 Fig — (A) LIM 1215 cells were treated with PPEA polyplex, polyIC, ZEGFR 1907’ affibody, or the cytotoxic drug WEHI-7326 for 4 days. (B) LIM 1215 cells were treated with PPEA polyplex, polyIC, ZEGFR 1907’ affibody, or the cytotoxic drug WEHI-7326 for 2 hr, then cultured for 4 days. (C) DiFi cells were treated with PPEA polyplex, polyIC, ZEGFR 1907’ affibody, or the cytotoxic drug WEHI-7326 for 2 hr, then cultured for 4 days. Cell proliferation was monitored using the CellTiter Glo assay. The cytotoxic drug WEHI-7326 was used as the positive control, i.e., WEHI-7326 induced 100% cell death. (TIFF) [file pone.0334584.s004.tiff]

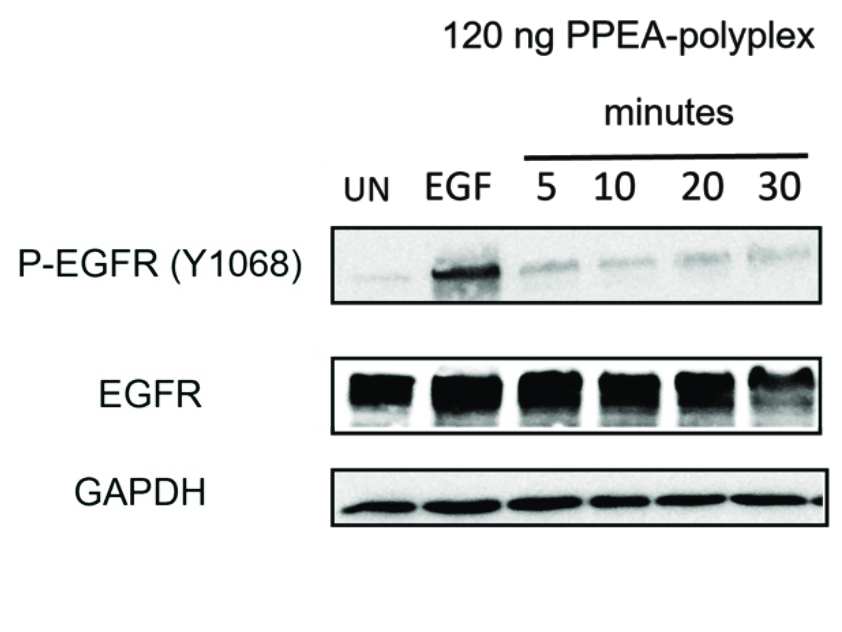

Supplement: S5 Fig — Cells were treated as described in “Materials and methods”. Untreated cells (UN) were used as negative control and cells incubated with EGF for 5 min were used as the positive control. (TIFF) [file pone.0334584.s005.tiff]

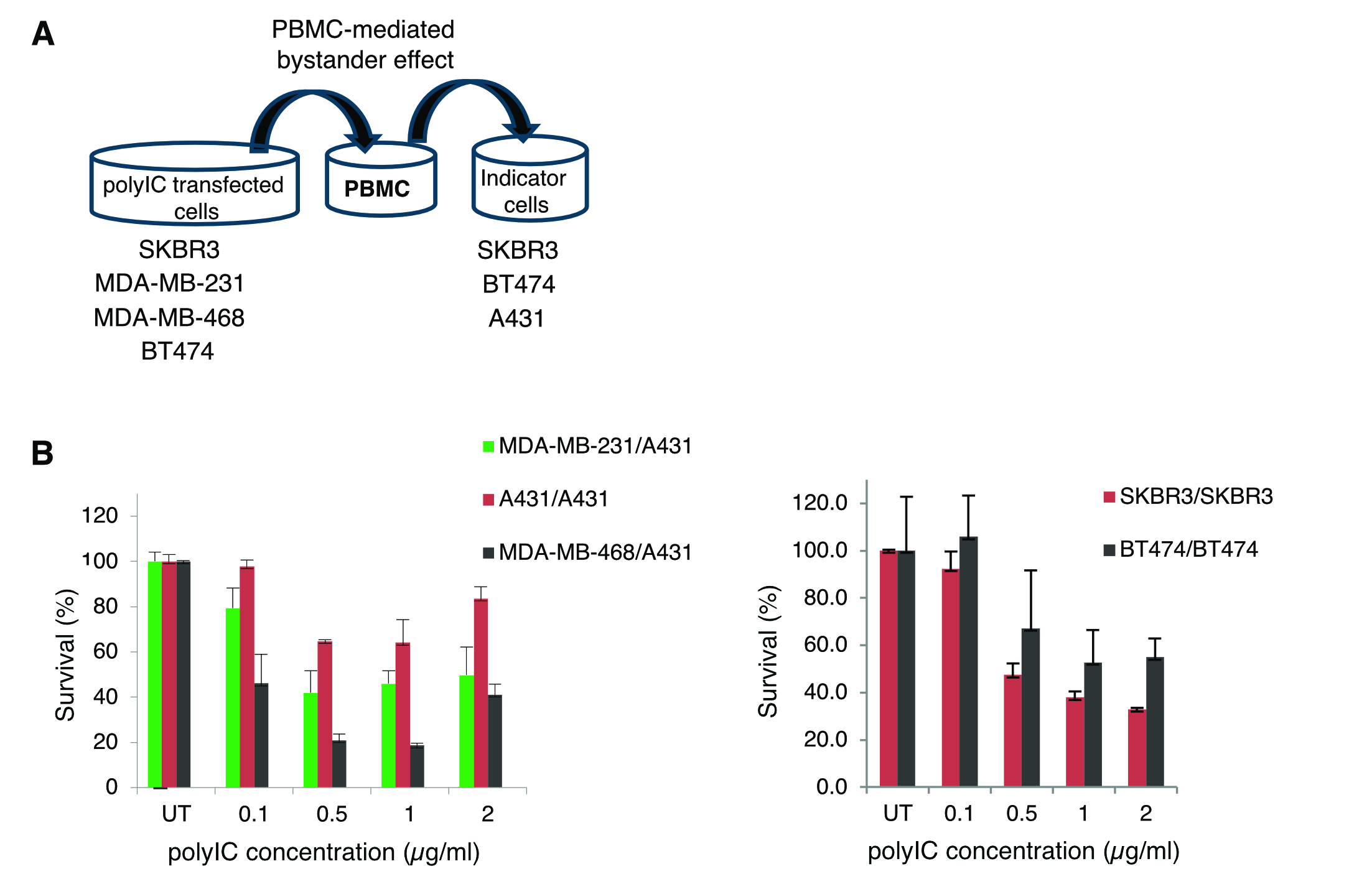

Supplement: S6 Fig — 30,000 A431, MDA-MB-231, MDA-MB-468, SKBR3 or BT474 cells were seeded into 48-well plates and grown overnight with 1 ml medium per well. Cells were then treated with PPEA-polyplex at the indicated concentrations. 24hrs after treatment 0.2 ml of medium from the treated cells (“conditioned medium”) was added to 3x10^5^ PBMCs/well, which had been seeded immediately following isolation into 96 well plates in 0.1 ml medium/well. Following 48 hrs incubation, 0.1 ml of medium from challenged PBMCs was then exchanged for 0.1 ml medium from non-treated cells (“indicator cells) seeded on 96 well plates (4000 cell/well), 24 hrs earlier. Survival of these cells was determined by methylene blue assay, 72 hrs after challenge with the medium from the PBMCs. (A) Shows experiment design; (B) Shows the PBMC-mediated bystander effect of PBMCs challenged with medium from polyIC treated MDA-MB-231, A431 and MDA-MB-468 cells on untreated A431 cells. (C) Shows the PBMC-mediated bystander effect of PBMCs challenged with medium from polyIC treated SKBR3 and BT474 cells on untreated SKBR3 and BT474 cells, respectively. (TIFF) [file pone.0334584.s006.tiff]

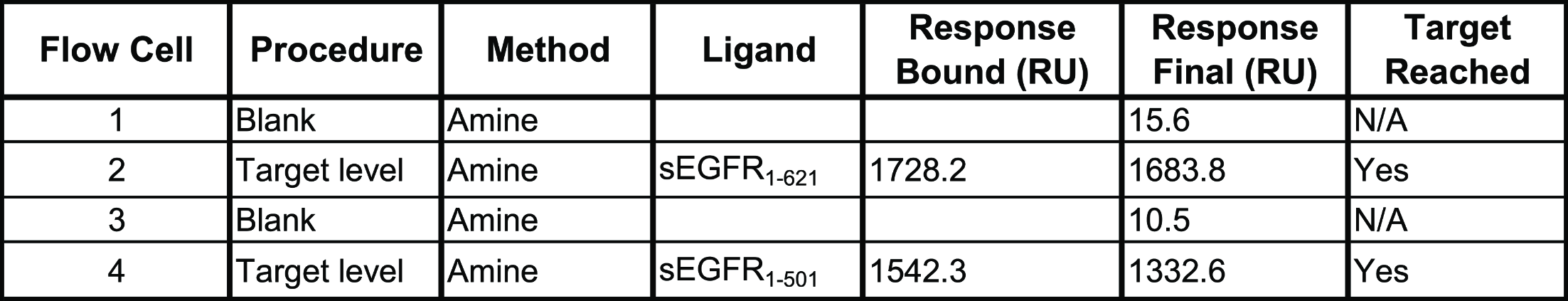

Supplement: S1 Table — (TIFF) [file pone.0334584.s007.tiff]

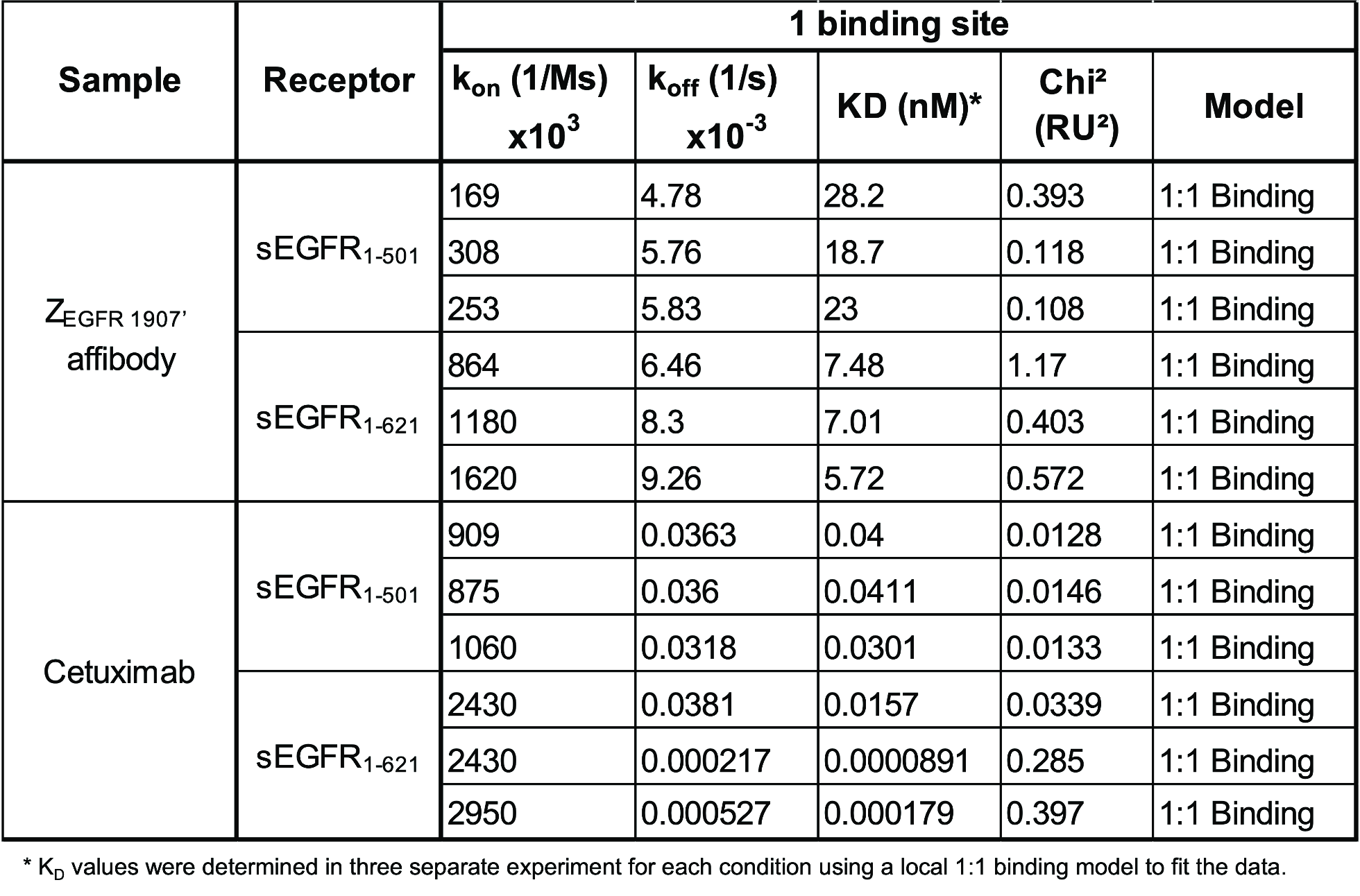

Supplement: S2 Table — (TIFF) [file pone.0334584.s008.tiff]

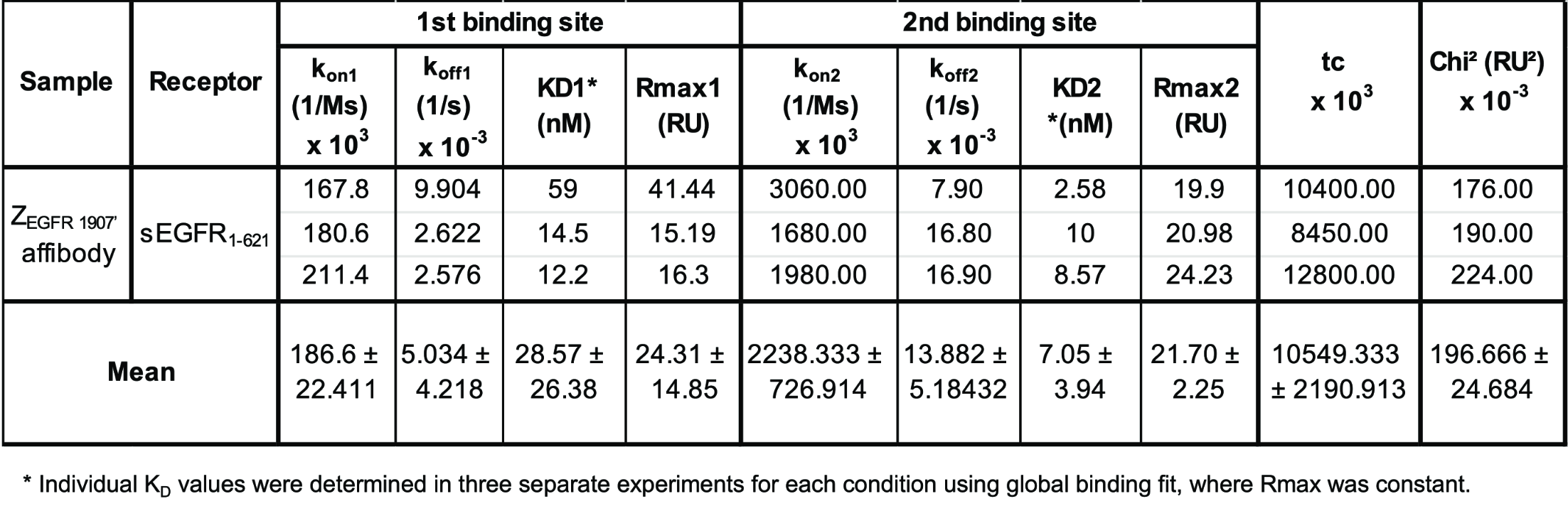

Supplement: S3 Table — (TIFF) [file pone.0334584.s009.tiff]
