# Supplementary material for: EGFR-targeted affibody–polyIC polyplex kills EGFR-overexpressing cancer cells without activating the EGFR
Source: PLoS One. 2026 May 5;21(5):e0334584. doi: 10.1371/journal.pone.0334584 (PMC13143101; doi:10.1371/journal.pone.0334584)
Supplement: Attachment — Submitted filename: Response to Reviewers.docx [file pone.0334584.s011.docx]

Dear Dr Aliabadi, 8^th^ January, 2026

On behalf of all of the authors, I would like to thank both the editors and the reviewers for the care with which they read and responded to our submission.

We believe our revised manuscript is improved and hope that it will meet with your approval for publication.

Yours sincerely

Antony Burgess

**Author Responses to the Editor and Reviewer Comments**

**Responses to Editor’s comments**

**“**Please make sure to address all the concerns raised by the three reviewers, especially the concerns reviewer 3 raised about the choice of the animal model and please explain how the EGFR targeted complex evaluated in this manuscript differs from or improves upon the EGF polyIC nanoparticle described in your recent publication.”

We have responded in detail to all the reviewers’ comments, explaining our use of the animal model and how the Affibody might have advantages over the EGF based polyplex.

**“**Please ensure that your manuscript meets PLOS ONE's style requirements, including those for file naming.”

We have followed the guidelines.

We will use the recommended form for our Data availability statement:

“All data are in the manuscript and/or supporting information files."

“You must submit original gel images”

We have included a file with the original gel images, one of the original images ( for the kinase

Blot, now S5 fig.) has been lost in the computer filing system. This figure was supporting information for similar data published previously by others. We have referenced their publications which show similar results to our own blot.

“ Please include your full ethics statement in the ‘Methods’ section of your manuscript file”

The ethics statement is now included in the Materials and Methods section.

“Supporting Information”

Captions for our Supporting Information are at the end of our manuscript.

There was a recommendation to cite specific previously published works, but that work did not seem relevant to our data, so we did not cite it. However, as a result of the reviewers’ comments, here was a need for several new citations and these have been included.

**Responses to Reviewers’ Comments**

**Reviewer 1**

**Point 1** We agree with the reviewer and we have revised the discussion to emphasize the differences between the affibody-based and EGF-based reagents.

**Point 2** a concise schematic or flow diagram summarizing the multi-step synthesis of LPEI-PEG-EGFR affibody conjugates – Now included as Supplementary Figure S1

**Point 3** Provide a mechanistic model summarizing how PPEA-polyplexes induce selective cytotoxicity The following paragraph has been added to the discussion:

“PolyIC is a potent activator of intracellular killing pathways as well as cytokine release(88-91). Delivering polyIC selectively to cancer cells provides the potential for a specific anti-tumour response (91,92). Many common cancers over-express the EGFR, by attaching polyIC to PEG and the poly-imine there is less non-specific cellular uptake; the addition of the high affinity EGFR ligand (i.e. the affibody) then directs the polyIC to the tumor cells. It was surprising to see the polyIC internalized without receptor kinase activation, so it will be intriguing to unravel the mechanism of internalization. Autocrine stimulation of EGF-HER3 oligomers is one possible mechanism, but detailed experiments will be required to discover the mechism of the internalization of the affibody-polyplex. Once in the endosome, the LPEI component will facilitate the disruption of the membrane and the entry of the polyIC to the cytoplasm, where both the dsRNA killing mechanisms and the induction of anti-tumor cytokines are initiated.”

**Point 4** Use more recent citations: We have added several recent citations which report the anti-tumor activity of polyIC. These references should give readers a pathway to exploring more about the potential of selectively delivered polyIC-complexes as anti-tumor agents.

**Reviewer 2**

**Point 1** The reviewer makes the point that we have used a variety of cell lines to study the different aspects of this project. In many cases the cell lines were chosen to allow us to study a the effects of the polyplexes on cells with different levels of EGFR, in others it was to measure immune responses and cell killing. We have described the use of each cell line clearly and at no stage did we select a result because it was the only cell which responded. The assays were performed in triplicate and with the large number of different cell lines we investigated for the assays, we believe the diversity and range add to the robustness of the results. Adding even more cell lines is unlikely to alter the conclusions to be drawn from our results.

**Point 2** The reviewer makes a valid point. We did not measure and report the EGFR levels for four of the cell lines MCF-7, MDA-MB-231, SK-BR-3 and BT-747. We have added a paragraph to the results which now reports the EGFR levels for these cell lines. “We also used three cell lines which express very low EGFR levels: BT-747(61), MCF-7(61) and SK-BR-3(61). Although we did not measure the EGFR levels on these cell lines each has been studied extensively and the flow cytometry data from Stanley, et al(61) shows they all have low EGFR levels. The other cell line used for the cytotoxicity studies MDA-MB-231 had low to moderate levels of the EGFR(61), i.e. EGFR was detectable by flow cytometry, but clearly the EGFR levels were only 5% the levels in the over-expressing cell line MDA-MB-468 (61).” Reference 61 is a paper by Stanley et al, 2017 which documents the receptor levels for many cell lines

**Point 3 Stats for appropriate experiments** Where possible we have added stats for appropriate experiments.

**Point 4** We have changed the title of the section” Abbreviations” to “Abbreviations and Terminology”. In this section we describe or definitions of low, moderate and high levels of EGFR on cell lines.

**Point 5** Several cell lines have undetectable levels of EGFR including SW620, U138MG and MCF-7 cells

**Point 6** The reviewer is correct: we have replaced “transfected” with “treated” throughout the manuscript.

**Point 7** The affibody is a protein purified from bacterial extracts. We know it binds with high affinity to the human EGFR, but we did not investigate its binding to the murine EGFR.

**Point 8** In the absence of polyIC, we did not observe toxicity from the affibody in mice.

**Point 9** The author is correct the heading for the animal experiment included Allografts. We do not present the results of any allograft experiments in the paper, so “and Allografts” has been removed for the sub-heading in the Materials and Methods section

**Pont 10** In response to the reviewer’s suggestion, we have added “As a control, we treated the animals with a PPEA-poly Inosine (polyI)-polyplex, made in the same way as the PPEA-polyIC-polyplex. Poly Inosine is not a double-stranded RNA and would not be expected to either kill the cells directly or to induce the immune cytokines which amplify the killing of the tumor cells”

**Points 11 and 12** The reviewer is correct we have extrapolated too far from our results. Our abstract and the discussion of the xenograft result have been revised accordingly.

**Point 13** The limitations of the in vitro data and the mouse model are pointed out in the new discussions.

**Point 14** All of the supplementary figures are now referred to as S1, S2, … etc

**Point 15** On Figures 4 and S2C the x-axes cannot be seen. These figures have been revised – the x-axes are now visible

**Point 16 “** MilliQ” has been replaced with “de-ionized water”.

**Point 17** The abbreviation sEGFR is now explained in the” Abbreviations and Terminology “ section.

**Point 18** The reviewer notes: In the Materials and Methods, not all cell lines list their origin (e.g. breast cancer**)**. We have revised the manuscript so when a cell line is first mentioned the tissue of origin is clear. Also the materials and methods includes the sentence “. Also we note that the materials and methods specifies “All cell lines were grown in their recommended media (DMEM from Gibco, RPMI 1640 from Sigma and EMEM from ATCC) supplemented with 10%FCS, -/+ Adds (1.08% thioglycerol, 50 mg/ml hydrocortisone, 100 U/ml insulin), -/+ G418.”

**Point 19**. For the cell viability assay, please include a supplementary table with the cell densities used. This density data is now included in the Materials and Methods

**Point 20** The reviewer correctly notes that Bortezomib is listed as one of two general cell death reagents in the Materials and Methods. Actually in this manuscript, only WEHI-7326 was used and the results are documented in Supplementary Figure S3. All cell lines tested in this study were killed by WEHI-7326. Bortezomib has been removed from the ” Materials and Methods” section.

**Point 21** The Ethics statement re euthanasia has been re-written

**Reviewer #3**

**General Comments**

We have added comments to the introduction and discussion to emphasize the difference between the EGF-based polyplex and the affibody-based polyplex. In particular, that the tumor killing by the affibody does not require that it activates the EGFR kinase. The observation that the killing of cell lines is dependent on the presence of the EGFR, but that the sensitivity is not related to the cell surface EGFR levels is new. This observation is likely to be important for predicting which patients are most likely to respond to these EGFR directed polyIC polyplexes. We do not have the data to determine the mechanism underlying the extreme sensitivity of some of the cell lines (e.g. the BT-20 Breast cancer cells), but in the discussion we present several possible models, including autocrine activation, EGFR hetero-oligomerization with the erbB3 system as well as alterations in the polyIC killing pathways. Significant resource, experimentation and time will be required to determine which mechanisms are responsible and will form the basis of future studies, but they are beyond the scope of this report.

Where necessary **we have revised the Tables and Figures** to bring them to publication quality and in accord with the PlosOne requirement.

As requested **we have revised the manuscript to improve nomenclature** consistency, remove typographical errors and mistakes in grammar.

**Specific comments**

**Point 1** FACS has been replaced by Flow cytometric throughout the manuscript

**Point 2** We have tried to ensure the Table formatting is robust and that text does not wrap around

Point 3 The reviewer is correct Figure 2C and Supplemental Figure S2B were the same. We have removed Supplemental figure S2B and made the appropriate changes in the figure legend and the text in the main manuscript.

**Point 4** The term “FACS” has been removed from the figure and the term Flow cytometry is now used in the legend for Figure 3

**Point 5** Reviewer: “Recommend including the EGFR expression”. The cell surface EGFR is almost certainly the critical data, but the total levels of EGFR may be an important indicator of the mechanism underlying the different sensitivities of the cell lines to PPEA. We prefer to leave that data in the manuscript figures, rather that relegate it to the supplementary data.

**Point 6** Reviewer: “ Figure 3B incorrectly states…”, the reviewer is correct the figure has been corrected

**Point 7** As recommended for Figure 4A, U87MGWT has been changed to U87MGWTEGFR

**Point 8** The reviewer makes the point “The data shown here is not sufficient to demonstrate that the PPEA poly IC polyplex inhibits cell growth since cell viability was only measured at one time point”. The reviewer is correct, indeed the assay as presented only presents a value proportional to the cell number – there may have been both proliferation and cell death, inhibition of proliferation or induction of cell death. Previous experience with the polyIC system, other assays at multiple times allows us to believe that the number of cells in the population is reduced in the presence of the PPEA, thus it is reasonable to describe the effects of PPEA as growth inhibition. The underlying reason for the growth inhibition is almost certainly a combination of induced apoptosis and reduced proliferation, but we chose not to dissect the causes in this study.

**Point 9** The experiment suggested by the reviewer would add definitively to the data, but the cells were generated in Melbourne and the cytokine assays were performed in Jerusalem. Exchanging the materials and expertise for this experiment would have been difficult and time consuming. We believe overall the data in our manuscript is sufficient support for the concept that the PPEA requires the EGFR for its cytokine inducing activities.

**Point 10** “Why were A431 cells and not U138-MG-WTEGFR cells used for this experiment?” Similarly to point 9 – we did not exchange the U87MGWTEGFR cells between the teams. Only the U87MG cell were used in this experiment. The legend to Figure 7 (now Figure 6) has been corrected, i.e. the U87MGWTEGFR cells have been removed.

**Point 11** The reviewer is correct, the title has been redrafted appropriately

**Point 12** Table 3 and Table 4 . The reviewer is correct, we can only say that the PPEA-polyplex was required for inhibiting the cells, not the polyIC or PPEA independently. We have altered the discussion to make this clear. Again the reviewer is correct, if we had used the PPEA-polyplex polyI in this experiment, we could be more definitive about the role of polyIC.

**Point 13** “Figure 7 and Supplemental Figure S5:” recommends moving data from S5 to Figure 7.

The supplementary figure S5 (now S6) has three parts and would overwhelm the chemotaxis data (now Figure 6). We think the results are clearer when Figure 6 only shows the chemotaxis data, so we have not combined these figures.

**Point 14** The reviewer suggests we should use an immunocompetent mouse model, A431 cells growing in a nude mouse model is not ideal, especially when the these mice lack the appropriate T-cell responses. Of course, the reviewer is correct, in an ideal world we should have used a mouse tumour cells, which express medium or high levels of the EGFR, growing in a mouse syngeneic host. The editor also requested that we should provide a response about the suitability of this mouse model However, mouse tumour cells do not often express high levels of the EGFR, e.g. the 4T1 cells referred to by the reviewer only express low levels of the mouse EGFR (*Nisticò, N.; Aloisio, A.; Lupia, A.; Zimbo, A.M.; Mimmi, S.; Maisano, D.; Russo, R.; Marino, F.; Scalise, M.; Chiarella, E.; et al. Development of Cyclic Peptides Targeting the Epidermal Growth Factor Receptor in Mesenchymal Triple-Negative Breast Cancer Subtype. Cells* ***2023****, 12, 1078.* [*https://doi.org/10.3390/cells12071078*](https://doi.org/10.3390/cells12071078)). Using the - mouse colon tumor MC38 (Tan, M.H., Holyoke, E.D. and Goldrosen, M.H., 1976. Murine colon adenocarcinomas: methods for selective culture in vitro. *Journal of the National Cancer Institute*, *56*(4), pp.871-873, which has low levels of mouse EGFR, we made several MC38-based cell lines which expressed medium or high levels of human EGFR-GFP. These cell lines did grow in syngeneic mice (C57Bl/6), but these cells cause ulceration which interfered with the tumor growth experiments. A further complication was associated with the loss of expression of the human EGFR-GFP from the MC38 cells when they were passaged. We agree a syngeneic mouse tumor model where the tumor cells express medium or high levels of the mouse EGFR, should give more information on the potential of affibody-polyplex as a cancer therapeutic, but a new mouse model needs to be developed. It is possible that 4T1 cells would be suitable for creating cell lines which express human EGFR-GFP and which would then grow in syngeneic mice, but that would require significantly new experimentation. It is still not clear that stable expression of the human EGFR could be achieved in that cell line. If the mouse EGFR was overexpressed in mouse cells, it would be necessary check that the affinity of the affibody for the mouse EGFR would be high enough for targeting. Our difficulties with the syngeneic mouse model involved considerable experimentation, but in the end the results did not improve our knowledge of the efficacy of the affibody-polyplex.

**Point 15** “Cetuximab is referred to as Erbitux” – recommend only use cetuximab not Erbitux. We have made this change

**Point 16** The reviewer is correct “killing effect in breast cell lines” should be changed to “killing effect in breast cancer cell lines” –we have made this change.

**Point 17** Please include line and page numbers. We have done this for the revised version of the manuscript

**Point 18** The manuscript needs to be edited prior too the submission of the revision. We will read the manuscript carefully, remove the typos and improve the grammar
