# Supplementary material for: EGFR-targeted affibody–polyIC polyplex kills EGFR-overexpressing cancer cells without activating the EGFR
Source: PLoS One. 2026 May 5;21(5):e0334584. doi: 10.1371/journal.pone.0334584 (PMC13143101; doi:10.1371/journal.pone.0334584)
Supplement: Attachment — Submitted filename: Response to Reviewers comments on our First Revision.docx [file pone.0334584.s012.docx]

Author responses to the Reviewers’ comments on the first revision of our manuscript

**Reviewer #1**

Was satisfied with our revision of the manuscript.

**Reviewer #2**

1. Indicated that some of our figures in the .pdf were pixelated and impossible to read. We have fixed this issue and believe all of the figures are now clear and easy to read.
2. We have attended to the problems with the statistics – all the details of the cell viability assay statistics are now included.
3. We have added the description of the statistical analysis method for the *in vivo* assay to the legend for Figure 8:

“Statistical analysis: Two-tailed paired t-test. Data are presented as mean ± SEM. P-values ware for comparison with the tumors growing in untreated (UT) mice; ** p < 0.01; *** p < 0.001.”

Reviewer #3

1. The reviewer indicated “: In this revised manuscript, the authors have provided additional text in the introduction to explain the rationale for using an affibody-based PPEA polyplex in addition to their prior work that utilized an EGF-based complex. Figure quality and readability have been improved, and several typos and grammatical errors have been corrected.”

Since Reviewer #2 still had some issues with our figures, so we have made sure these will be clear and easy to read.

2. The reviewer states “. The biggest concern that remains is that the authors present in vitro data that the PPEA polyplex induces anti-tumor activity via an immune-mediated mechanism. However, they used an in vivo model that lacks an immune system, which demonstrated that PPEA polyplex induces anti-tumor activity via a non-immune mediated mechanism.” The reviewer adds “As the manuscript stands, their in vitro and in vivo data appear to be contradictory.”

Our in vitro and in vivo data are complementary – we observe killing in vitro without T-cell effects and we observe killing in vivo without T-cell effects. PolyIC is know to induce tumor cell killing once internalized, both directly and via cytokine activation of innate immunity via dendritic cells, NK cells, macrophages, eosinophils and neutrophils. In vitro the killing is essentially via the direct induction of apoptosis, although there maybe some indirect jilling through cytokines such as TNF-α. In vivo, there was direct killing, but the innate immune system of the nude mice is functional and also likely to kill of the tumor cells. There is no T-cell immune activation in the nude mice, so this means of extra targeting would not occur in our mouse model, but in syngeneic systems such as occurs in the clinical situation, not only would the direct and innate killing be induced, there should be T-cell tumor cytotoxicity as well. The ability of polyIC to kill human tumour cells in nude mice has been documented many times.

For example Talmadge, J. E., Adams, J., Phillips, H., Collins, M., Lenz, B., Schneider, M., Schlick, E., Ruffmann, R.,Wiltrout, R. H., and Chirigos, M. A.(1985). Immunomodulatory effects in mice of polyinosinic-polycytidylic acid complexed with poly-L-lysine and carboxymethylcellulose. Cancer Res. 45, 1058–1065

Kumar, H., Koyama, S., Ishii, K. J.,Kawai, T., and Akira, S. (2008). Cutting edge: cooperation of IPS-1- and TRIF-dependent pathways in polyIC-enhanced antibody production and cytotoxic T cell responses. J. Immunol. 180, 683–687.

Okada, H., Kalinski, P., Ueda, R., Hoji, A., Kohanbash, G., Donegan, T. E., Mintz, A. H., Engh, J. A., Bartlett, D. L., Brown, C. K., Zeh, H., Holtzman, M. P., Reinhart, T. A., Whiteside, T. L., Butterﬁeld, L. H., Hamilton, R. L., Potter, D. M., Pollack, I. F., Salazar, A. M., and Lieberman, F. S. (2011). Induction of CD8+ T-cell responses against novel glioma-associated antigen peptides and clinical activity by vaccinations with {alpha}-type 1 polarized dendritic cells and polyinosinic-polycytidylic acid stabilized by lysine and carboxymethyl cellulose

in patients with recurrent malignant glioma. J. Clin. Oncol. 29,330–336.

Vidit Singh, Anna Chernatynskaya, Lin Qi, Hsin-Yin Chuang, Tristan Cole, Vimalin Mani Jeyalatha, Lavanya Bhargava, W. Andrew Yeudall, Laszlo Farkas, and Hu Yang (2024)Liposomes-Encapsulating Double-Stranded Nucleic Acid (Poly I:C) for Head and Neck Cancer Treatment *ACS Pharmacology & Translational Science* *7* (5), 1612-1623DOI: 10.1021/acsptsci.4c00121

We acknowledge that the nude mouse model we used is not perfect, but it does show the ability of the PPEA-polyplex to reach the tumor, for the polyIC to be internalized and for the internalized polyIC to induce the killing of the tumor cells *in vivo*. These results indicate that the PPEA-polyplex is likely to be a potent anti-tumor agent in vivo and when an intact immune system is present the agent is likely to be even more effective. The suggested experiments with a syngeneic mouse tumor model would likely yield more information, but there are many difficulties and the syngeneic tumor model we tried led to ulceration in the mice. We could not justify attempting to reduce the side-effects. We believe that the readers will recognize the value of our results and the limitations of the nude mouse model.

3. The reviewer wrote “Additionally, while the authors present data that show differential response to PPEA polyplex based on EGFR expression, they only present data for EGFR surface expression for 3 of the cell lines used in the study, despite requests from two reviewers to provide data for additional cell lines.”

We had measured the surface EGFR levels for many more cell lines; figure 3B has been expanded to include the EGFR surface expression levels for 16 cell lines. We used a smaller number of cell lines for the targeting studies, but our cytotoxicity results were encouraging and along with our cytokine release studies and cell activation studies, we believe our results are a strong indication of the potential of this reagent for cancer therapy trials.

4. The reviewer states “Lastly, since their point-by-point response did not include the reviewer comments, I was unable to fully ascertain if all comments from reviewers 1 and 2 were addressed” From the responses of reviewers 1 and 2 – they were almost entirely satisfied with this revision – above we have responded to reviewer #2 two comments.

**Specific comments:**

1. Page 21 The reviewer is correct. We now only reference Supplementary Figure 5 once and we have improved the sentence structure.
2. Table 3 “In the results section of the text (page 22 line 12), the authors incorrectly state that these are pro-inflammatory cytokines.” Despite this comment, there is an extensive literature indicating that IP10 and Gro-α are pro-inflammatory cytokines. For example:

**IP 10**

Zhang Z, Kaptanoglu L, Tang Y, Ivancic D, Rao SM, Luster A, Barrett TA, Fryer J. IP-10-induced recruitment of CXCR3 host T cells is required for small bowel allograft rejection. Gastroenterology. 2004 Mar;126(3):809-18. doi: 10.1053/j.gastro.2003.12.014. PMID: 14988835

Dufour JH, Dziejman M, Liu MT, Leung JH, Lane TE, Luster AD (April 2002). ["IFN-gamma-inducible protein 10 (IP-10; CXCL10)-deficient mice reveal a role for IP-10 in effector T cell generation and trafficking"](https://doi.org/10.4049%2Fjimmunol.168.7.3195). *Journal of Immunology*. **168** (7): 3195-204. Bibcode:2002JImm..168.3195D. doi:10.4049/jimmunol.168.7.3195. PMID 1190 7072.

**Gro-alpha**

Ichikawa A, Kuba K, Morita M, Chida S, Tezuka H, Hara H, Sasaki T, Ohteki T, Ranieri VM, dos Santos CC, Kawaoka Y, Akira S, Luster AD, Lu B, Penninger JM, Uhlig S, Slutsky AS, Imai Y. CXCL10-CXCR3 enhances the development of neutrophil-mediated fulminant lung injury of viral and nonviral origin. Am J Respir Crit Care Med. 2013 Jan 1;187(1):65-77. doi: 10.1164/rccm.201203-0508OC. Epub 2012 Nov 9. PMID: 23144331; PMCID: PMC3927876.

Consequently, we have decided to retain the term pro-inflammatory cytokines for these molecules.

3. The reviewer states “Page 23 lines 22 and 29, Table 4. The authors also incorrectly use the term cytotoxic cytokines when referring to pro-inflammatory cytokines.” It is correct that these are pro-inflammatory cytokines, but they are also referred to as cytotoxic cytokines. Foe example:

**TNF-alpha**

Li M, Beg AA. Induction of necrotic-like cell death by tumor necrosis factor alpha and caspase inhibitors: novel mechanism for killing virus-infected cells. J Virol. 2000 Aug;74(16):7470-7. doi: 10.1128/jvi.74.16.7470-7477.2000. PMID: 10906200; PMCID: PMC112267.

Gregory T. Baxter, Richard C. Kuo, Orla J. Jupp, [Peter Vandenabeele](https://www.sciencedirect.com/author/7005733218/peter-vandenabeele), [David J. MacEwan](https://www.sciencedirect.com/author/7005028554/david-j-macewan) (1999)

Tumor Necrosis Factor-α Mediates Both Apoptotic Cell Death and Cell Proliferation in a Human Hematopoietic Cell Line Dependent on Mitotic Activity and Receptor Subtype Expression Journal of Biological Chemistry, [Volume 274, Issue 14](https://www.jbc.org/issue/S0021-9258(19)X8083-7), p9539-9547,

<https://doi.org/10.1074/jbc.274.14.9539>

Ksontini R, MacKay SLD, Moldawer LL. Revisiting the Role of Tumor Necrosis Factor α and the Response to Surgical Injury and Inflammation. *Arch Surg.* 1998;133(5):558–567. doi:10.1001/archsurg.133.5.558

In the context of our manuscript we believe that the description of IFNγ and TNFα as cytotoxic cytokines is accurate and we have decided to retain that nomenclature.

**IFN-gamma**

Badie B, Schartner J, Vorpahl J, Preston K. Interferon-gamma induces apoptosis and	augments the expression of Fas and Fas ligand by microglia in vitro. Exp Neurol. 2000	Apr;162(2):290-6. doi: 10.1006/exnr.1999.7345. PMID: 10739635.

Fang C, Weng T, Hu S, Yuan Z, Xiong H, Huang B, Cai Y, Li L, Fu X. IFN-γ-induced ER stress impairs autophagy and triggers apoptosis in lung cancer cells. Oncoimmunology. 2021 Aug 10;10(1):1962591. doi: 10.1080/2162402X.2021.1962591. PMID: 34408924; PMCID: PMC8366549.

4. The reviewer states “The formatting of the tables at the end of the manuscript still makes them difficult to read.” In our first revision we did adjust the Tables to improve the formatting, but the conversion to the .pdf format was still not satisfactory. We have made further adjustments to make sure they format well in the .pdf.
5. The reviewer is correct - All of the inappropriate “Flow”s have now been changed to “flow”s
6. The reviewer is correct - All of the references to transfection, transfected and transfecting have now been replaced with treatment, treated and treating.

We are grateful for the opportunity to work with the editors and the reviewers to improve our manuscript. We believe we have responded to all the comments in an appropriate way and hope that our manuscript is now suitable for publication in PlosOne

Yours sincerely

Antony Burgess
