# Supplementary material for: EGFR-targeted affibody–polyIC polyplex kills EGFR-overexpressing cancer cells without activating the EGFR
Source: PLoS One. 2026 May 5;21(5):e0334584. doi: 10.1371/journal.pone.0334584 (PMC13143101; doi:10.1371/journal.pone.0334584)
Supplement: Attachment — Submitted filename: Responses to Reviewer comments on our second Revision.docx [file pone.0334584.s013.docx]

Author Responses to Final comments by Reviewer 3

In response to the reviewer’s final concern regarding the use of the nude mouse model and the mechanism of action of polyIC in vivo, we have revised the Discussion to explicitly address these points. Specifically, we clarify that although nude mice lack T cells, they retain functional innate immune components, including NK cells, macrophages, dendritic cells and other effector cells, which can contribute to tumor cell killing.

We have also expanded the Discussion to describe the mechanism of action of polyIC following intracellular delivery, including direct tumor cell killing via dsRNA-induced apoptotic pathways and activation of innate immune responses through Toll-like receptor 3 (TLR3) and cytosolic sensors such as melanoma differentiation–associated protein 5 (MDA5).

Appropriate citations have been added to support these additions, in particular clarifying the mechanism by which polyIC mediates its anti-tumor activity in the absence of adaptive immunity.
